# Predictive Binding Affinity of Plant-Derived Natural Products Towards the Protein Kinase G Enzyme of *Mycobacterium tuberculosis* (*Mt*PknG)

**DOI:** 10.3390/plants8110477

**Published:** 2019-11-06

**Authors:** Rana M. Qasaymeh, Dino Rotondo, Carel B. Oosthuizen, Namrita Lall, Veronique Seidel

**Affiliations:** 1 Strathclyde Institute of Pharmacy and Biomedical Sciences, University of Strathclyde, Glasgow G4 0RE, UK; rana-mohammad-mahmoud-qasaymeh@strath.ac.uk (R.M.Q.); d.rotondo@strath.ac.uk (D.R.); 2 Department of Plant and Soil Sciences, University of Pretoria, Pretoria 0002, South Africa; u04405765@tuks.co.za (C.B.O.); namrita.lall@up.ac.za (N.L.); 3 School of Natural Resources, University of Missouri, Columbia, MO 65211, USA; 4 College of Pharmacy, JSS Academy of Higher Education and Research, Mysuru, Karnataka 570015, India

**Keywords:** AutoDock Vina, flavonoids, molecular docking, *Mycobacterium tuberculosis*, *Pelargonium reniforme*, *Pelargonium sidoides*, Protein kinase G (PknG), SiteMap

## Abstract

Tuberculosis (TB), caused by *Mycobacterium tuberculosis*, is a growing public health concern worldwide, especially with the emerging challenge of drug resistance to the current drugs. Efforts to discover and develop novel, more effective, and safer anti-TB drugs are urgently needed. Products from natural sources, such as medicinal plants, have played an important role in traditional medicine and continue to provide some inspiring templates for the design of new drugs. Protein kinase G, produced by *M. tuberculosis* (*Mt*PKnG), is a serine/threonine kinase, that has been reported to prevent phagosome-lysosome fusion and help prolong *M. tuberculosis* survival within the host’s macrophages. Here, we used an in silico, target-based approach (docking) to predict the interactions between *Mt*PknG and 84 chemical constituents from two medicinal plants (*Pelargonium reniforme* and *Pelargonium sidoides*) that have a well-documented historical use as natural remedies for TB. Docking scores for ligands towards the target protein were calculated using AutoDock Vina as the predicted binding free energies. Ten flavonoids present in the aerial parts of *P. reniforme* and/or *P.* *sidoides* showed docking scores ranging from −11.1 to −13.2 kcal/mol. Upon calculation of all ligand efficiency indices, we observed that the (−ΔG/MW) ligand efficiency index for flavonoids (**4**), (**5**) and (**7**) was similar to the one obtained for the AX20017 control. When taking all compounds into account, we observed that the best (−ΔG/MW) efficiency index was obtained for coumaric acid, coumaraldehyde, *p*-hydroxyphenyl acetic acid and *p*-hydroxybenzyl alcohol. We found that methyl gallate and myricetin had ligand efficiency indices superior and equal to the AX20017 control efficiency, respectively. It remains to be seen if any of the compounds screened in this study exert an effect in *M. tuberculosis*-infected macrophages.

## 1. Introduction

Tuberculosis (TB), an infectious disease which mainly affects the lungs and is caused by the bacterium *Mycobacterium tuberculosis*, has plagued humans since antiquity [1]. In 2017, the World Health Organisation estimated that there were 10 million TB cases worldwide, which resulted in a mortality rate of 1.6 million. The treatment of TB necessitates complex drug regimens, with adverse effects and interactions, and is associated with poor patient compliance. This has led to the evolution of multidrug-resistant (MDR-TB) and extensively drug-resistant (XDR-TB) strains. Patients with MDR- or XDR-TB require a lengthy course of a combination of drugs that are more expensive, more toxic, and not always effective [2]. With the continuous increase in the number of drug-resistant TB cases, it is vital to identify drugs that could inhibit new druggable targets in *M. tuberculosis* [3,4,5,6,7,8]. Eleven different serine/threonine protein kinases have been reported in mycobacteria, including protein kinase G in *M. tuberculosis* (*Mt*PknG), which is of particular interest, not only because it regulates the signal transduction pathways that control the metabolism of *M. tuberculosis*, but because it plays an essential role in promoting the survival and persistence of this pathogen within macrophages. *Mt*PknG is a soluble enzyme, secreted by *M. tuberculosis,* that belongs to the family of prokaryotic Ser/Thr protein kinases (STPKs). The latter play an important function in the phosphorylation of proteins involved in signal transduction pathways that control a range of metabolic processes in bacteria. *Mt*PknG is essential for sustaining TB infection, by promoting the survival and persistence of *M. tuberculosis* within infected macrophages through blocking phagosome–lysosome fusion. It has recently been identified as a key regulator in the mycobacterial metabolism of carbon and nitrogen. Additionally, it is required for the formation of mycobacterial biofilms and is involved in the development of anti-TB drug resistance in mycobacteria. Targeting *Mt*PknG represents one possible approach for the discovery of new anti-TB drugs [9,10,11,12,13,14,15,16,17,18,19,20,21,22,23].

Products of natural origin contain a unique pool of incredibly chemically diverse molecules that have specifically evolved to interact with biological targets and have already provided some invaluable leads for drug design [24]. Plant-based medicines, in particular, are used widely by traditional healers in different parts of the world, including for the treatment of TB and TB-related symptoms [25,26,27,28]. Many plant extracts and plant-derived chemicals have demonstrated antitubercular activity [29,30,31,32]. *Pelargonium sidoides* DC. and *Pelargonium reniforme* Curtis (Geraniaceae) are two plants indigenous to South Africa that are used as natural remedies for wound-healing (aerial parts), as well as for gastrointestinal disorders, persistent coughs and respiratory tract infections, including TB (roots) [33,34]. A tincture made from *P. sidoides* and *P. reniforme* roots, known as “Umckaloabo”, was introduced into Europe in the early 20th century by the Englishman Charles Stevens, who claimed to have recovered from TB after taking “Umckaloabo”. The remedy, known as “Stevens’ Consumption Cure”, was subsequently used in Europe to treat more than 800 TB patients and is currently licensed for the treatment of upper respiratory tract infections [34,35,36,37,38,39]. The exact nature and mechanism of action of the substance(s) responsible for the effect observed in TB cases have yet to be fully understood. 

Molecular docking is an in silico, target-based approach used in the virtual screening of small molecules (ligands) against a given protein (target) [40], that has already been applied to the search for new anti-TB drugs from natural sources [41,42]. Here, we report on the use of a guided docking approach, using AutoDock Vina, to predict the interactions between some natural products from the roots/aerial parts of *P. reniforme/P. sidoides* and *Mt*PknG, as a starting point in the search for new anti-TB agents. 

## 2. Results

A total of eighty-four natural products from the aerial parts and roots of both *Pelargonium* spp. were selected for our molecular docking study. They were grouped into four categories, namely phenolics, coumarins (comprising coumarin glycosides and coumarin sulfates), flavonoids, and other miscellaneous compounds. The tetrahydrobenzothiophene derivative AX20017, a known inhibitor of the target enzyme, was retrieved from its co-crystallised complex with *Mt*PknG, and re-docked as a control against the enzyme to validate the docking conditions [43]. The binding site of the co-crystallised inhibitor was identified as the most favourable docking site, with a higher site score and druggability score (1.138 and 1.174, respectively) when compared to the other potential binding sites (1.027 and 1.034) (Table 1). Knowing the nature of the key amino acid residues involved in the binding [43], we employed a rigid ligand docking approach to predict the affinity of each natural product from *Pelargonium* towards *Mt*PknG. The docking scores obtained using Auto Dock Vina ranged between −5.8 and −13.2 kcal/mol. The docking score for the AX20017 control was −7.9 kcal/mol (RMSD to input ligand = 0.5476 Å) (Table S1). Ten flavonoids present in the aerial parts of *P. reniforme* and/or *P. sidoides* showed docking scores ranging from −11.1 to −13.2 kcal/mol (Table 2). Ligand efficiency indices were calculated for all ligands and are presented in Table 2 and Table S1.

**Figure 1:**
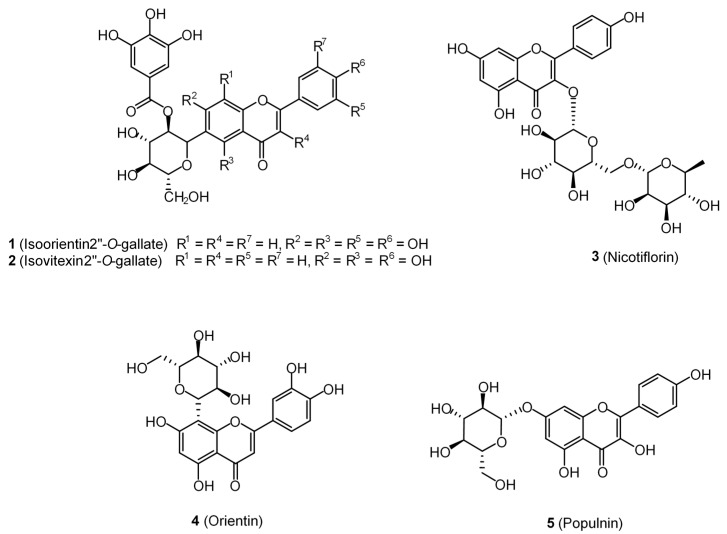
Structures of *Pelargonium* flavonoids (**1–5**).

**Figure 2:**
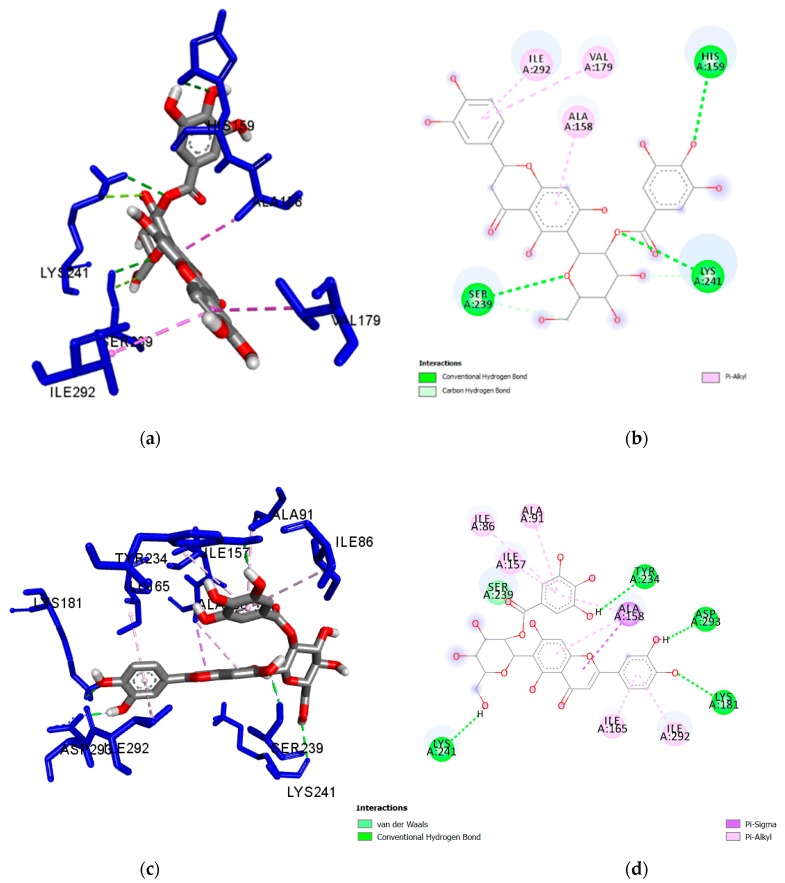
(**a**) Docked pose of rigid isoorientin 2″-*O*-gallate (**1**) in the *Mt*PknG binding site, showing molecular interactions—hydrogen and hydrophobic bonds as green and pink/purple dashed lines, respectively; (**b**) 2D plot of interactions between (**1**) and key residues of *Mt*PknG generated by BIOVIA Discovery Studio visualizer. The solvent accessible surface is depicted as a background grey circle with the radius proportional to the exposure. (**c**) Docked pose of flexible isoorientin 2″-*O*-gallate (**1**) in the *Mt*PknG binding site showing molecular interactions—hydrogen and hydrophobic bonds as green and pink/purple dashed lines, respectively; (**d**) 2D plot of interactions between (**1**) and key residues of *Mt*PknG generated by BIOVIA Discovery Studio visualizer. The solvent accessible surface is depicted as a background grey circle with the radius proportional to the exposure.

**Figure 3:**
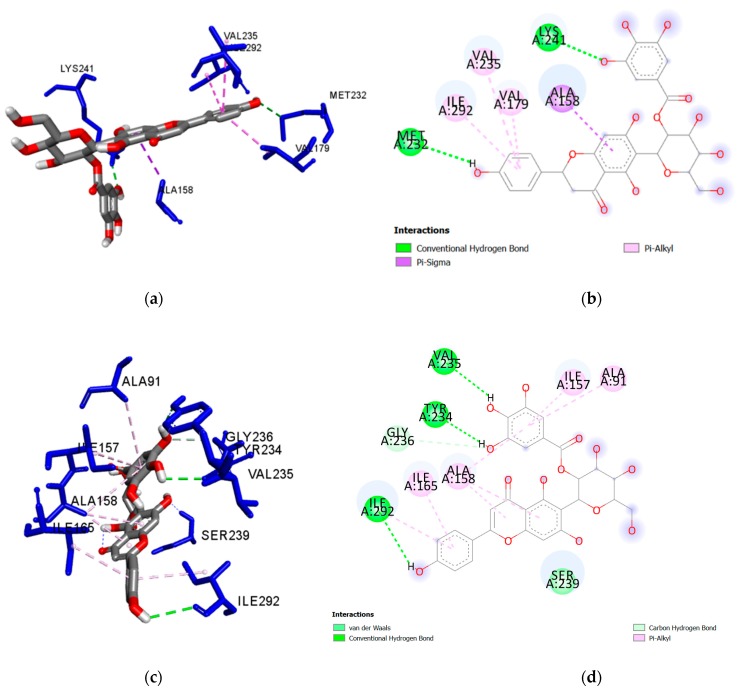
(**a**) Docked pose of rigid isovitexin 2″-*O*-gallate (**2**) in the *Mt*PknG binding site, showing molecular interactions—hydrogen and hydrophobic bonds as green and pink/purple dashed lines, respectively; (**b**) 2D plot of interactions between (**2**) and key residues of *Mt*PknG generated by BIOVIA Discovery Studio visualizer. The solvent accessible surface is depicted as a background grey circle with the radius proportional to the exposure. (**c**) Docked pose of flexible isovitexin 2″-*O*-gallate (**2**) in the *Mt*PknG binding site showing molecular interactions—hydrogen and hydrophobic bonds as green and pink/purple dashed lines, respectively; (**d**) 2D plot of interactions between (**2**) and key residues of *Mt*PknG generated by BIOVIA Discovery Studio visualizer. The solvent accessible surface is depicted as a background grey circle with the radius proportional to the exposure.

The nature of the intermolecular interactions formed with the amino acid residues of *Mt*PknG were further investigated for the five flavonoid ligands showing the strongest docking scores, namely isoorientin 2″-*O*-gallate (**1**), isovitexin 2″-*O*-gallate (**2**), nicotiflorin (**3**), orientin (**4**) and populnin (**5**) (Figure 1) (Table 3). A closer look at the interactions between isoorientin 2″-*O*-gallate (**1**) and *Mt*PknG revealed that the sugar moiety of this flavonoid was bound via strong hydrogen bonds to Ser239 and Lys241, while the para-hydroxyl group of the gallate unit was bound to His159. This specific hydrogen-bonding network enabled the flavone backbone of (**1**) to be positioned in such a way as to develop further hydrophobic interactions with Ala158, Val179 and Ile292 (Figure 2a,b). In the case of isovitexin 2″-*O*-gallate (**2**), the gallate unit and the para-hydroxyl on the B ring of the flavonoid formed hydrogen bonds with Lys241 and Met232 (2.168 and 2.903 Å, respectively). Hydrophobic interactions were also present between (**2**) and Ala158, Val179, Val235 and Ile292 (Figure 3a,b). Nicotiflorin (**3**) showed numerous interactions with *Mt*PknG, including strong hydrogen bonds (contact distances < 2.5 Å) with Glu233, Glu280, Gln238 and Ser 239 (Figure S1a). The B-ring hydroxyl groups of orientin (**4**) showed three hydrogen bonds with Lys181, and one with Asp293, while the flavone backbone interacted via hydrophobic interactions with Ala158 and Ile157 (Figure S2a). Populnin (**5**) also interacted strongly through hydrogen bonds with Lys181, Asp 293 and Gln238 (2.498, 2.213 and 2.278 Å, respectively) and via hydrophobic interactions with Ala158, Ile157, Ile165, Ile292 and Met283 (Figure S3a). An overlay of the docked poses of the control inhibitor AX20017, isoorientin 2″-*O*-gallate (**1**) and isovitexin 2″-*O*-gallate (**2**) in the *Mt*PknG binding site is presented in Figure S4. 

Owing to the presence of several rotatable bonds in the five aforementioned flavonoids, a flexible ligand docking approach was further employed, to identify differences between poses obtained by flexible and rigid docking (Table 4). Specific molecular interactions between *Mt*PknG and isoorientin 2″-*O*-gallate (**1**), isovitexin 2″-*O*-gallate (**2**), nicotiflorin (**3**), orientin (**4**) and populnin (**5**) are depicted in Figure 2c,d, Figure 3c,d, Figures S1b, S2b, and S3b, respectively.

## 3. Discussion

*Mt*PknG is a multidomain protein that comprises an N-terminal rubredoxin-like domain (including two thioredoxin motifs), followed by a central kinase domain (containing the ATP-binding site) and, finally, a C-terminal tetratricopeptide-repeat domain. The N-terminal domain is crucial for the kinase activity of *Mt*PknG. The C-terminal domain acts as a regulator of such activity by stabilizing interactions with the substrates [21,43,44]. *Mt*PknG shares a low sequence similarity with human STPKs and the binding pocket of its enzymatic active site contains a unique set of amino acid residues, that does not occur in any human kinase. This makes *Mt*PknG an interesting target, that can be exploited in the development of novel selective inhibitors [16,17,43,44].

The tetrahydrobenzothiophene compound AX20017 interacts with the ATP binding pocket of *Mt*PknG via a unique set of hydrophobic amino acids, comprising Ile165, Val179, Gly236 and Ile292 of the ATP-binding site, and Ile87 and Ala92 of the N-terminal region. Other interacting residues include Ala158, Lys181, Met232, Glu233, Val235 and Asp293 [11,43,45]. Other potential inhibitors of *Mt*PknG, identified through molecular docking screenings, and of natural origin, include withanolide derivatives from the ayurvedic medicinal plant *Withania somnifera* (L.) Dunal [46] and the marine-derived sclerotiorin (IC_50_ = 76.5 μM) [47]. These compounds have demonstrated interactions with Glu233 and Val235, Gly237, Gln238 and Ser239, Lys241, Ile292, Ser293, Ala158, Ile165, Val179, Lys181, Met232, Ile292, Asp293 [46], and with Gly161, Leu162 and Lys278, respectively [47].

As observed in the control inhibitor AX20017 and the withanolide derivatives, compound (**1**) interacted with key amino acid residues of the *Mt*PknG active site, i.e., Lys 241, Ser239, Ala158 and Ile292, in both rigid and flexible docking. Also as observed in AX20017 and the withanolides, it displayed a further interaction with Val179 in rigid docking, whereas, in flexible docking, it interacted with Ile165 and Asp293. It also interacted with Lys181 (as for AX20017) in flexible docking. Compound (**2**) interacted with Ala158, Ile292 and Val235 in both rigid and flexible docking, similar to the control inhibitor and the withanolide derivatives. In rigid docking alone, it also interacted with Lys241, Val179 and Met232 (as did the control inhibitor and the withanolide derivatives). In flexible docking, it interacted with Ser239 (as did the withanolides), Gly236 (similar to AX20017), and Ile165 (similar to both the control and the withanolides). Compound (**3**) interacted with Ser239, Ile86, Ile292, Ala158, Ile165 and Val235 in both rigid and flexible docking, similar to AX20017 and the withanolide derivatives. In rigid docking alone, it also interacted with Glu233 (as did the control and the withanolides) and Gln238 (as did the withanolides). In flexible docking, it further interacted with Lys181. Compound (**4**) interacted with Ala158 and Ile157 in both rigid and flexible docking. In rigid docking alone, it also interacted with Lys181 and Asp293 (as did the control inhibitor and the withanolides). In flexible docking, more interactions were observed with Glu233, Val235, Ser239, Ile165, Val179 and Ile292. Compound (**5**) interacted with Gln238, Lys181, Ile292, Ala158, Ile165 and Ile157, in both rigid and flexible docking. In rigid docking alone, it also interacted with Asp293 (similar to the control inhibitor and the withanolides). In flexible docking, an additional interaction with Ser239 (as seen for the withanolides) was observed. Overall, the interactions observed in flexible ligand and in rigid ligand docking protocols showed good agreement with previously published data [43,44,45,46].

In order to adequately compare the efficiency of smaller size ligands with larger size ligands, three ligand efficiency indices were further calculated for all compounds. These included a ligand efficiency index (coded as LE3 in Table 2 and Table S1) calculated by dividing the predicted free energy of binding (−ΔG) by the molecular weight (MW) for each compound [48]. We observed that this (−ΔG/MW) efficiency index for flavonoids (**4**), (**5**) and (**7**) was similar to the one obtained for the AX20017 control (LE3 = 0.03). When taking all compounds into account, we observed that the best (−ΔG/MW) efficiency index was obtained for coumaric acid, coumaraldehyde, *p*-hydroxyphenyl acetic acid and *p*-hydroxybenzyl alcohol (LE3 = 0.05)**.**

Previous studies on *Pelargonium* have revealed that extracts and constituents of *P. sidoides/reniforme* leaves possess moderate activity against Gram-positive and Gram-negative bacteria [49]. Extracts obtained from *Pelargonium* roots possess direct antimycobacterial activity, including activity against *M. tuberculosis* [33,50]. Some mixtures of long-chain fatty acids, active against rapidly growing mycobacteria, have been isolated from *P. sidoides/reniforme* root extracts, with linoleic acid identified as one of the active compounds [51]. Epigallocatechin and scopoletin, from *P. sidoides* roots, have exhibited activity against *M. smegmatis* [50]. The exact identity of the constituents responsible for the direct activity of *P. sidoides/reniforme* against *M. tuberculosis*, however, is less clear. No specific antitubercular constituent has so far been isolated from *P. reniforme* and, although a chemical analysis of a *P. sidoides* root extract, active on *M. tuberculosis*, found four coumarins (umckalin, scopoletin, 6,8-dihydroxy-5,7-dimethoxycoumarin, and 6,8-dihydroxy-7-methoxycoumarin) and two flavonoids (catechin and epigallocatechin), none of these compounds, when tested against *M. tuberculosis* and in *M. tuberculosis*-infected macrophages, have demonstrated any biological effects [50].

As early as 1930, it was suggested that the curative properties of both *Pelargonium* spp. in TB cases were likely to be caused by the stimulation of a macrophage-mediated killing of *Mycobacterium* [37]. Studies since then have reported that extracts and constituents of *P. sidoides*, in particular gallic acid and methyl gallate, could reduce the survival of the intracellular parasite *Leishmania donovani*, and this was attributed to the activation of some non-specific immune response mechanisms within macrophages [33,52,53]. A similar effect has been observed in *Candida albicans-* and *Listeria monocytogenes*-infected macrophages, treated with *P. sidoides* root extracts [54]. Evidence for the immunomodulatory role of *Pelargonium* root extracts in the presence of intracellular residing mycobacteria was observed when gallic acid, methyl gallate, myricetin and isoquercetin, were identified as the constituents from *P. reniforme* roots responsible for increasing the killing activity of *M. tuberculosis*-infected macrophages [55]. It this study, we found that only methyl gallate (LE3 = 0.04) and myricetin (LE3 = 0.03) had ligand efficiency indices that were superior and equal to the AX20017 control efficiency, respectively. Interestingly, it was also previously observed that nicotiflorin, rutin and *p*-coumaric acid had immunomodulatory activity [56,57]. It remains to be seen if any of the compounds tested in this study exert an effect in *M. tuberculosis*-infected macrophages.

## 4. Materials and Methods 

### 4.1. Protein Preparation

The three-dimensional crystal structure of the target *Mt*PknG protein (PDB ID:2PZI), in complex with its ligand inhibitor (AX20017), was retrieved from the RCSB Protein Data Bank (http://www.pdb.org). The protein was used as a rigid structure and all water molecules and hetero-atoms were removed using BIOVIA Discovery Studio Visualizer v.4.5 (Accelrys). A PDBQT file of the target protein, with added polar hydrogen atoms, was subsequently prepared using AutoDock Tools v. 1.5.6rc3 [58].

### 4.2. Ligand Preparation 

The ligands selected for docking were 84 natural products, previously isolated from the roots and the aerial parts of *P. reniforme* and *P. sidoides* [55,59,60,61,62]. All chemical structures were retrieved from SciFinder (https://scifinder.cas.org/scifinder/login). The structure of the ligand inhibitor (AX20017) was retrieved from its corresponding complex with *Mt*PknG (PDB ID:2PZI), using BIOVIA Discovery Studio Visualizer v.4.5 (Accelrys). Each ligand structure was exported to ChemOffice v.16.0, and geometry-optimised using MM2 energy minimisation [63]. Docking files for all ligands were then prepared, using AutoDock Tools v. 1.5.6rc3 [58]. All rotatable bonds present were treated as non-rotatable, to perform rigid docking and minimise standard errors (typically of 2.85 kcal/mol), likely due to ligands with many active rotatable bonds [64]. Gasteiger charges were assigned [65] and files were saved as PDBQT formats in preparation for docking.

### 4.3. Binding Site Analysis and Prediction

To analyse and identify the binding site and potential allosteric sites, SiteMap (Schrödinger, LLC, New York, NY, 2018) was utilised. This software employs van der Waals probes, in order to identify energetically favourable binding pockets. SiteMap was tasked to identify the five top-ranked possible receptor sites, using the default settings. The site score, druggability score and size were used to determine the most favourable receptor site [66,67].

### 4.4. Grid Box Preparation and Docking Studies 

Parameters for the grid box, to define the size of the searching space around the *Mt*PknG binding site residues, were prepared using AutoDock Tools v. 1.5.6rc3, while molecular docking simulations were executed with AutoDock Vina v. 1.1.2 [64]. The centre of the grid box was set to x = 19.234, y = −9.412, z = −3.495. Its size was 22 × 20 × 20 points in the x, y and z dimensions. The spacing was set at 1 Å. To validate the accuracy of the docking, and to allow a comparison between docking scores, the co-crystallised inhibitory ligand AX20017 was re-docked into *Mt*PknG. Different orientations of the ligands were searched and ranked based on their energy scores. Upon visual inspection of all binding poses obtained, only poses with the lowest root mean square deviation (RMSD) value (threshold < 1.00 Å) were considered to provide a high accuracy of docking. The default values set in Autodock Vina were used as the parameters for the rigid ligand docking (exhaustiveness = 8). The exhaustiveness was set to 16 for the flexible ligand docking. The docking scores were calculated as the predicted free energies of binding (ΔG in kcal/mol). The lowest binding free energy—i.e., best score for the docking pose with the lowest (RMSD)—indicated the highest predictive ligand/protein affinity. Ligand efficiency indices were also calculated for all ligands as the free energy of binding/number of heavy atoms (LE1= −ΔG/NHA), free energy of binding/number of carbons (LE2= −ΔG/NoC), and free energy of binding/molecular weight (LE3= −ΔG/MW) [48] (Table S1).

### 4.5. Protein–Ligand Interactions and Predictive Inhibition

Specific intermolecular interactions between the best ligand docking poses and the binding site of *Mt*PknG were further visualised using BIOVIA Discovery Studio Visualizer v.4.5 (Accelrys) (Table S2 and Figure 1 and Figure 2).

## 5. Conclusions

A molecular docking approach was conducted to predict the binding affinity of 84 natural products present in the aerial parts and/or roots of *Pelargonium reniforme* and *Pelargonium sidoides* for the mycobacterial enzyme *Mt*PknG. A total of ten flavonoids showed high docking scores and, among them, compounds (**4**), (**5**) and (**7**) exhibited a (−ΔG/MW) ligand efficiency index similar to the one obtained for the AX20017 control. A high ligand efficiency index was also observed for coumaric acid, coumaraldehyde, *p*-hydroxyphenyl acetic acid and *p*-hydroxybenzyl alcohol, methyl gallate and myricetin. Some of these compounds can be found in *Pelargonium* aerial parts, suggesting that the roots may not be the only plant part that could have anti-TB potential. In fact, the selection of *Pelargonium* roots over the aerial parts for use as an anti-TB remedy by traditional healers is customary, rather than intentional [68]. Further in vitro and in vivo studies are required to establish the effectiveness of these compounds in inhibiting *Mt*PknG and in controlling TB.

## Figures and Tables

**Table 1:** Identified binding sites for *Mt*PknG using SiteMap.

Binding Site	SiteScore ^1^	DScore ^2^	Volume (Å)
1 (AX20017-Co-crystallised site)	1.138	1.174	271.31
2	1.027	1.034	1548.65
3	1.012	1.067	270.97
4	0.950	0.971	301.84
5	0.940	0.968	498.38

^1^ Quality of the identified binding site (SiteScore = 0.0733 sqrt(n) + 0.6688 e − 0.20 p). ^2^ Druggability score.

**Table 2:** Origin of *Pelargonium* natural products (**1–10**), and their predicted free binding energy (docking score ΔG in kcal/mol) and ligand efficiency indices towards *Mt*PknG ^a^.

Compound	*P. reniforme*	*P. sidoides*	Docking Score	Ligand Efficiency Indices		
				LE1	LE2	LE3
Isoorientin 2″-*O*-gallate (**1**)	AP	AP	−13.2	0.31	0.47	0.02
Isovitexin 2″-*O*-gallate (**2**)		AP	−12.6	0.30	0.45	0.02
Nicotiflorin (**3**)	AP		−12.2	0.29	0.45	0.02
Orientin (**4**)	AP	AP	−11.8	0.37	0.56	0.03
Populnin (**5**)	AP		−11.6	0.36	0.55	0.03
Rutin (**6**)	AP		−11.4	0.27	0.42	0.02
Vitexin (**7**)	AP	AP	−11.2	0.36	0.53	0.03
Quercimeritrin (**8**)	AP		−11.2	0.34	0.53	0.02
Isoorientin (**9**)	AP	AP	−11.2	0.35	0.53	0.02
Glucoluteolin (**10**)		AP	−11.1	0.35	0.53	0.02

AP = Aerial parts; R = Roots. LE1 defines the ligand efficiency coefficient calculated as—(ΔG/number of heavy atoms in the ligand). LE2 defines the ligand efficiency coefficient calculated as—(ΔG/number of carbons in the ligand). LE3 defines the ligand efficiency coefficient calculated as—(ΔG/molecular weight of the ligand). ^a^ The re-docked AX20017 control inhibitor had a docking score of −7.9 kcal/mol against *Mt*PknG and ligand efficiencies of LE1, LE2 and LE3 of 0.44, 0.61 and 0.03, respectively.

**Table 3:** Detailed molecular interactions obtained following the rigid ligand docking of *Pelargonium* compounds (**1**) to (**5**), with *Mt*PknG.

Ligand	Interacting Residues	Distance (Å)	Category	Type
Isoorientin 2″-*O*-gallate (**1**)	Lys241	2.650	H-Bond	Conventional
	Ser239	2.825	H-Bond	Conventional
	His159	3.063	H-Bond	Conventional
	Lys241	3.140	H-Bond	Carbon Hydrogen Bond
	Ser239	3.512	H-Bond	Carbon Hydrogen Bond
	Ile292	4.701	Hydrophobic	Pi-Alkyl
	Val179	4.893	Hydrophobic	Pi-Alkyl
	Ala158	4.195	Hydrophobic	Pi-Alkyl
Isovitexin 2″-*O*-gallate (**2**)	Lys241	2.168	H-Bond	Conventional
	Met232	2.903	H-Bond	Conventional
	Ala158	3.898	Hydrophobic	Pi-Sigma
	Ile292	4.811	Hydrophobic	Pi-Alkyl
	Val235	5.002	Hydrophobic	Pi-Alkyl
	Val179	4.317	Hydrophobic	Pi-Alkyl
Nicotiflorin (**3**)	Glu233	2.134	H-Bond	Conventional
	Glu280	2.286	H-Bond	Conventional
	Gln238	2.290	H-Bond	Conventional
	Ser239	2.357	H-Bond	Conventional
	Ile86	5.025	Hydrophobic	Alkyl
	Ile292	3.768	Hydrophobic	Pi-Sigma
	Ile292	3.898	Hydrophobic	Pi-Sigma
	Ile157	4.605	Hydrophobic	Pi-Alkyl
	Ala91	4.608	Hydrophobic	Pi-Alkyl
	Ala158	4.846	Hydrophobic	Pi-Alkyl
	Ala158	5.218	Hydrophobic	Pi-Alkyl
	Ile165	5.290	Hydrophobic	Pi-Alkyl
	Met283	5.468	Hydrophobic	Pi-Alkyl
	Val235	5.471	Hydrophobic	Pi-Alkyl
Orientin (**4**)	Lys181	2.248	H-Bond	Conventional
	Lys181	2.715	H-Bond	Conventional
	Lys181	2.669	H-Bond	Conventional
	Asp293	2.728	H-Bond	Conventional
	Ala158	4.835	Hydrophobic	Pi-Alkyl
	Ala158	4.453	Hydrophobic	Pi-Alkyl
	Ile157	4.846	Hydrophobic	Pi-Alkyl
Populnin (**5**)	Asp293	2.213	H-Bond	Conventional
	Gln238	2.278	H-Bond	Conventional
	Lys181	2.498	H-Bond	Conventional
	Gln238	3.455	H-Bond	Carbon Hydrogen Bond
	Ile292	3.872	Hydrophobic	Pi-Sigma
	Ala158	4.526	Hydrophobic	Pi-Alkyl
	Ala158	4.714	Hydrophobic	Pi-Alkyl
	Ile165	4.817	Hydrophobic	Pi-Alkyl
	Met283	5.127	Hydrophobic	Pi-Alkyl
	Ile165	5.150	Hydrophobic	Pi-Alkyl
	Ile292	5.159	Hydrophobic	Pi-Alkyl
	Ile157	5.311	Hydrophobic	Pi-Alkyl

**Table 4:** Detailed molecular interactions obtained following the flexible ligand docking of *Pelargonium* compounds (**1**) to (**5**), with *Mt*PknG.

Ligand	Interacting Residues	Distance (Å)	Category	Type
Isoorientin 2″-*O*-gallate (**1**)	Lys181	2.583	H-Bond	Conventional
	Lys241	2.657	H-Bond	Conventional
	Ser239	2.086	H-Bond	Conventional
	Tyr234	2.022	H-Bond	Conventional
	Asp293	1.867	H-Bond	Conventional
	Ile 86	5.361	Hydrophobic	Pi-Alkyl
	Ala158	3.929	Hydrophobic	Pi-Sigma
	Ile292	5.263	Hydrophobic	Pi-Alkyl
	Ala91	4.738	Hydrophobic	Pi-Alkyl
	Ile165	4.592	Hydrophobic	Pi-Alkyl
	Ala158	4.984	Hydrophobic	Pi-Alkyl
	Ile157	5.154	Hydrophobic	Pi-Alkyl
	Ala158	5.213	Hydrophobic	Pi-Alkyl
Isovitexin 2″-O-gallate (**2**)	Ser239	2.184	H-Bond	Conventional
	Tyr234	2.241	H-Bond	Conventional
	Val235	2.699	H-Bond	Conventional
	Ile292	3.044	H-Bond	Conventional
	Gly236	3.376	H-Bond	Carbon Hydrogen Bond
	Ala158	3.914	Hydrophobic	Pi-Alkyl
	Ile292	4.878	Hydrophobic	Pi-Alkyl
	Ile165	4.373	Hydrophobic	Pi-Alkyl
	Ala158	4.793	Hydrophobic	Pi-Alkyl
	Ala91	4.847	Hydrophobic	Pi-Alkyl
	Ile157	5.105	Hydrophobic	Pi-Alkyl
	Ala158	5.089	Hydrophobic	Pi-Alkyl
Nicotiflorin (**3**)	Lys181	3.005	H-Bond	Conventional
	Ser239	2.146	H-Bond	Conventional
	Asn281	2.163	H-Bond	Conventional
	Val235	2.174	H-Bond	Conventional
	Ile292	3.747	Hydrophobic	Pi-Sigma
	Ile86	4.966	Hydrophobic	Alkyl
	Val235	5.072	Hydrophobic	Pi-Alkyl
	Ile292	4.468	Hydrophobic	Pi-Alkyl
	Ala158	5.195	Hydrophobic	Pi-Alkyl
	Ile165	4.364	Hydrophobic	Pi-Alkyl
	Ile165	5.392	Hydrophobic	Pi-Alkyl
Orientin (**4**)	Ile157	2.477	H-Bond	Conventional
	Glu233	2.407	H-Bond	Conventional
	Val235	2.155	H-Bond	Conventional
	Val235	2.423	H-Bond	Conventional
	Gly237	2.227	H-Bond	Conventional
	Ser239	2.379	H-Bond	Conventional
	Glu280	2.411	H-Bond	Conventional
	Ala158	3.574	Hydrophobic	Pi-Sigma
	Ala158	3.885	Hydrophobic	Pi-Alkyl
	Ile165	4.567	Hydrophobic	Pi-Alkyl
	Ile165	5.400	Hydrophobic	Pi-Alkyl
	Val179	4.437	Hydrophobic	Pi-Alkyl
	Ile292	5.460	Hydrophobic	Pi-Alkyl
	Ile292	4.538	Hydrophobic	Pi-Alkyl
Populnin (**5**)	Gln238	2.130	H-Bond	Conventional
	Gln238	2.443	H-Bond	Conventional
	Ser239	2.297	H-Bond	Conventional
	Asn281	2.296	H-Bond	Conventional
	Lys181	2.699	H-Bond	Conventional
	Lys181	2.571	H-Bond	Conventional
	Ala158	4.391	Hydrophobic	Pi-Alkyl
	Ile165	5.080	Hydrophobic	Pi-Alkyl
	Ile292	5.175	Hydrophobic	Pi-Alkyl
	Ile157	4.571	Hydrophobic	Pi-Alkyl
	Ala158	4.148	Hydrophobic	Pi-Alkyl
	Ile165	4.783	Hydrophobic	Pi-Alkyl
	Ile292	5.122	Hydrophobic	Pi-Alkyl

## Supplementary Materials

Supplementary materials can be found at https://www.mdpi.com/2223-7747/8/11/477/s1. Figure S1: Docked pose of nicotiflorin (3) in the *Mt*PknG binding site showing molecular interactions—hydrogen-bonds as green dashed lines and hydrophobic bonds as pink/purple dashed lines—between (3) and *Mt*PknG, generated by BIOVIA Discovery Studio visualizer, Figure S2: Docked pose of orientin (4) in the *Mt*PknG binding site showing molecular interactions—hydrogen-bonds as green dashed lines and hydrophobic bonds as pink/purple dashed lines—between (4) and *Mt*PknG, generated by BIOVIA Discovery Studio visualizer, Figure S3: Docked pose of populnin (5) in the *Mt*PknG binding site showing molecular interactions—hydrogen-bonds as green dashed lines and hydrophobic bonds as pink/purple dashed lines—between (5) and *Mt*PknG, generated by BIOVIA Discovery Studio visualizer, Table S1: Origin of *Pelargonium* natural products and their predicted free binding energy (docking score ΔG in kcal/mol) and ligand efficiency indices towards *Mt*PknG.
