# Supplementary material for: Predictive Binding Affinity of Plant-Derived Natural Products Towards the Protein Kinase G Enzyme of Mycobacterium tuberculosis (MtPknG)
Source: Plants (Basel). 2019 Nov 6;8(11):477. doi: 10.3390/plants8110477 (PMC6918344; doi:10.3390/plants8110477)
Supplement: Supplementary file 1 [file plants-08-00477-s001.pdf]

Supplementary materials

Predictive Binding Affinity of plant-Derived Natural Products Towards the Protein Kinase G Enzyme of *Mycobacterium tuberculosis* (*Mt*PknG)

Rana M. Qasaymeh [1], Dino Rotondo [1], Carel B. Oosthuizen [2], Namrita Lall [2,3,4] and Veronique Seidel [1,*]

[1] Strathclyde Institute of Pharmacy and Biomedical Sciences, University of Strathclyde, Glasgow G4 0RE, UK; rana-mohammad-mahmoud-qasaymeh@strath.ac.uk; d.rotondo@strath.ac.uk; veronique.seidel@strath.ac.uk
[2] Department of Plant and Soil Sciences, University of Pretoria, Pretoria 0002, South Africa; u04405765@tuks.co.za; namrita.lall@up.ac.za;
[3] School of Natural Resources, University of Missouri, Columbia, MO 65211, United States
[4] College of Pharmacy, JSS Academy of Higher Education and Research, Mysuru, Karnataka 570015,, India.
* Correspondence: veronique.seidel@strath.ac.uk; Tel.: +44-141-548-2751 (V.S.)

Received: 10 October 2019; Accepted: 31 October 2019; Published: date

Table 1. Origin of *Pelargonium* natural products and their predicted free binding energy (docking score ΔG in kcal/mol) and ligand efficiency indices towards *Mt*PknG[a].

**Compound**	***P. reniforme***	***P. sidoides***	**Docking Score**	**Ligand Efficiency Indices**		
Phenolics						
				LE1	LE2	LE3
Shikimic acid 3,5-di-*O*-gallate	AP		-10.7	0.31	0.51	0.02
(α,β)-3,4-Di-*O*-galloylglucopyranoside	AP		-10.6 (α)	0.31	0.53	0.02
			-10.0 (β)	0.29	0.50	0.02
Salidroside-6''-*O*-gallate	AP		-10.2	0.32	0.46	0.02
Glucogallin	AP	AP	-9.3	0.40	0.72	0.03
Shikimic acid 3-*O*-gallate	R	R	-9.1	0.40	0.65	0.03
p-coumaroyl-4-*O*-β -D-glucoside	AP		-9.1	0.40	0.61	0.03
Gallic acid butyl ester	AP		-8.3	0.52	0.75	0.04
Glycerol-1-gallate	AP		-8.2	0.48	0.82	0.03
Caffeic acid	R		-7.6	0.58	0.84	0.04
Ethyle gallate	AP	AP	-7.6	0.54	0.84	0.04
Ferulic acid	R		-7.5	0.54	0.75	0.04
p-coumaric acid	R		-7.4	0.62	0.82	0.05
p-coumaraldehyde	R		-7.0	0.64	0.78	0.05
Methyl gallate	AP, R	AP, R	-6.9	0.53	0.86	0.04
p-hydroxyphenyl acetic acid	AP		-6.9	0.63	0.86	0.05
p-hydroxybenzyl alcohol	AP		-6.7	0.74	0.96	0.05
Vanillic acid	R		-6.6	0.55	0.83	0.04
Protocatechuic acid	R		-6.5	0.59	0.93	0.04
p-hydroxybenzoic acid	R		-6.2	0.62	0.89	0.04
p-hydroxyphenyl ethanol	AP		-5.8	0.58	0.73	0.04

AP= Aerial parts; R = Roots.

LE1 defines the ligand efficiency coefficient calculated as -(ΔG/number of heavy atoms in the ligand). LE2 defines the ligand efficiency coefficient calculated as -(ΔG/number of carbons in the ligand). LE3 defines the ligand efficiency coefficient calculated as -(ΔG/molecular weight of the ligand).

[a]The re-docked AX20017 control inhibitor had a docking score of -7.9 kcal/mol against *Mt*PknG and ligand efficiencies LE1, LE2 and LE3 of 0.44, 0.61 and 0.03, respectively.

Table 1. (Cont.). Origin of *Pelargonium* natural products and their predicted free binding energy (docking score ΔG in kcal/mol) and ligand efficiency indices towards *Mt*PknG[a].

Compound	*P. reniforme*	*P. sidoides*	Docking Score	Ligand Efficiency Indices		
Coumarins						
				LE1	LE2	LE3
8-hydroxy-5,7-dimethoxycoumarin-6-sulfate		R	-8.8	0.42	0.80	0.04
Magnolioside		R	-8.7	0.35	0.54	0.02
5,6-dimethoxycoumarin-7-sulfate		R	-8.7	0.44	0.79	0.04
7-hydroxycoumarin-6,8-bisulfate		R	-8.7	0.40	0.97	0.02
7-methoxycoumarin-6,8-bisulfate		R	-8.7	0.38	0.87	0.02
6-hydroxy-5,7-dimethoxycoumarin-8-sulfate		R	-8.5	0.40	0.77	0.04
7-hydroxy-5,6-dimethoxycoumarin-8-sulfate		R	-8.4	0.40	0.76	0.04
6,7-dihydroxycoumarin-8-sulfate		R	-8.3	0.46	0.92	0.03
Isofraxoside		R	-8.3	0.32	0.52	0.02
5,6,7,8-tetramethoxycoumarin (Artelin)		R	-8.2	0.43	0.63	0.03
7,8-dihydroxycoumarin-6-sulfate		R	-8.2	0.46	0.91	0.03
6-methoxycoumarin-7-sulfate		R	-8.2	0.46	0.82	0.03
8-hydroxy-7-methoxycoumarin-6-sulfate		R	-8.1	0.43	0.81	0.03
5,6-dihydroxy-7-methoxycoumarin (Isofraxetin)	R		-7.9	0.53	0.79	0.04
7,8-dihydroxy-5,6-dimethoxycoumarin		R	-7.9	0.46	0.72	0.03
7-hydroxy-6-methoxycoumarin-8-sulfate		R	-7.8	0.41	0.78	0.03
8-hydroxy-5,6,7-trimethoxycoumarin	R	R	-7.7	0.43	0.64	0.03
7,8-dihydroxy-6-methoxycoumarin (Fraxetin)		R	-7.7	0.51	0.77	0.04
6,7,8-trihydroxycoumarin	R	R	-7.7	0.55	0.86	0.04
7-acetoxy-5,6-dimethoxycoumarin		R	-7.6	0.40	0.58	0.03
6,8-dihydroxy-7-methoxycoumarin		R	-7.6	0.51	0.76	0.04
8-hydroxy-6,7-dimethoxycoumarin (Fraxidin)	R		-7.5	0.47	0.68	0.03
7- hydroxy-5,6-dimethoxycoumarin (Umckalin)		R	-7.5	0.47	0.68	0.03
6,8-dihydroxy-5,7-dimethoxycoumarin		R	-7.5	0.44	0.68	0.03
Umckalin-7-β-D-glucoside		R	-7.5	0.28	0.44	0.02
5,6,7-trimethoxycoumarin		R	-7.4	0.44	0.62	0.03
6-hydroxy-5,7-dimethoxycoumarin (Fraxinol)	R		-7.4	0.46	0.67	0.03
7- hydroxy-6-methoxycoumarin (Scopoletin)	R	R	-7.3	0.52	0.73	0.04

AP= Aerial parts; R = Roots.

LE1 defines the ligand efficiency coefficient calculated as -(ΔG/number of heavy atoms in the ligand). LE2 defines the ligand efficiency coefficient calculated as -(ΔG/number of carbons in the ligand). LE3 defines the ligand efficiency coefficient calculated as -(ΔG/molecular weight of the ligand).

[a]The re-docked AX20017 control inhibitor had a docking score of -7.9 kcal/mol against *Mt*PknG and ligand efficiencies LE1, LE2 and LE3 of 0.44, 0.61 and 0.03, respectively.

Table 1. (Cont.). Origin of *Pelargonium* natural products and their predicted free binding energy (docking score ΔG in kcal/mol) and ligand efficiency indices towards *Mt*PknG[a].

Compound	*P. reniforme*	*P. sidoides*	Docking Score	Ligand Efficiency Indices		
Flavonoids						
				LE1	LE2	LE3
Isoorientin 2″-*O*-gallate	AP	AP	-13.2	0.31	0.47	0.02
Isovitexin 2″-*O*-gallate		AP	-12.6	0.30	0.45	0.02
Kaempferol 3-*O*-β-D-rutinoside (Nicotiflorin)	AP		-12.2	0.29	0.45	0.02
Orientin	AP	AP	-11.8	0.37	0.56	0.03
Kaempferol 7-*O*-β-D-glucoside (Populnin)	AP		-11.6	0.36	0.55	0.03
Quercetin 3-*O*-β-D-rutinoside (Rutin)	AP		-11.4	0.27	0.42	0.02
Quercetin 7-*O*-β-D-glucoside (Quercimeritrin)	AP		-11.2	0.34	0.53	0.02
Isoorientin	AP	AP	-11.2	0.35	0.53	0.02
Vitexin	AP	AP	-11.2	0.36	0.53	0.03
Luteolin-7-*O*-β-D-glucoside (Glucoluteolin)		AP	-11.1	0.35	0.53	0.02
Isovitexin	AP	AP	-10.4	0.34	0.50	0.02
Kaempferol-3-*O*-β-D-glucoside (Astragalin)	R		-10.3	0.32	0.49	0.02
Myricetin	R		-10.2	0.44	0.68	0.03
Quercetin		AP	-9.9	0.45	0.66	0.03
Orientin 2″-*O*-gallate	AP	AP	-9.9	0.23	0.35	0.02
Naringenin-7-*O*-β-D-glucoside (Prunin)	AP		-9.8	0.32	0.47	0.02
Quercetin-3-*O*-β-D-glucoside (Isoquercetin)	R		-9.8	0.30	0.47	0.02
Kaempferol-3-*O*-β-D-galactoside (Trifolin)	R		-9.7	0.30	0.46	0.02
Vitexin 2″-*O*-gallate		AP	-9.7	0.23	0.35	0.02
Taxifolin-3-*O*-β-D-glucoside		AP	-9.7	0.29	0.46	0.02
Myricetin-3-*O*-β-D-glucoside (Isomericitrin)	R		-9.4	0.28	0.45	0.02
Dihydrokaempferol 3-*O*-β-D-glucoside		AP	-9.2	0.29	0.44	0.02
Taxifolin-7-*O*-β-D-glucoside	AP		-9.2	0.28	0.44	0.02
Epigallocatechin-3-*O*-gallate		AP	-9.2	0.28	0.42	0.02
Gallocatechin	R	R	-8.5	0.39	0.57	0.03
Afzelechin	R		-8.1	0.41	0.54	0.03
Catechin	R	R	-8.1	0.39	0.54	0.03
Dihydroquercetin (Taxifolin)	AP		-8.0	0.36	0.53	0.03

Dihydrokaempferol (Aromadendrin)	AP	-7.9	0.38	0.53	0.03

AP= Aerial parts; R = Roots.

LE1 defines the ligand efficiency coefficient calculated as -(ΔG/number of heavy atoms in the ligand). LE2 defines the ligand efficiency coefficient calculated as -(ΔG/number of carbons in the ligand). LE3 defines the ligand efficiency coefficient calculated as -(ΔG/molecular weight of the ligand).

[a]The re-docked AX20017 control inhibitor had a docking score of -7.9 kcal/mol against *Mt*PknG and ligand efficiencies LE1, LE2 and LE3 of 0.44, 0.61 and 0.03, respectively.

Table 1. (Cont.). Origin of *Pelargonium* natural products and their predicted free binding energy (docking score ΔG in kcal/mol) and ligand efficiency indices towards *Mt*PknG[a].

Compound	*P. reniforme*	*P. sidoides*	Docking Score	Ligand Efficiency Indices		
**Miscellaneous**						
				LE1	LE2	LE3
β-sitosterol	R	R	-10.3	0.34	0.36	0.02
Phyllantusiin E	AP		-10.1	0.48	0.78	0.03
Brevifolin carboxylic acid	AP		-10.0	0.48	0.77	0.03
Phyllantusiin E *O*-methyl ester	AP		-9.2	0.42	0.66	0.03
Reniformin	R		-9.1	0.27	0.34	0.02
β-sitosterol-3-*O*-β-D-glucoside	R		-8.4	0.20	0.24	0.01
4,6-Dihydroxyacetophenone 2-*O*-β-D-glucoside		AP	-7.6	0.33	0.54	0.02

AP= Aerial parts; R = Roots.

LE1 defines the ligand efficiency coefficient calculated as - (ΔG/number of heavy atoms in the ligand). LE2 defines the ligand efficiency coefficient calculated as - (ΔG/number of carbons in the ligand). LE3 defines the ligand efficiency coefficient calculated as - (ΔG/molecular weight of the ligand).

[a]The re-docked AX20017 control inhibitor had a docking score of -7.9 kcal/mol against *Mt*PknG and ligand efficiencies LE1, LE2 and LE3 of 0.44, 0.61 and 0.03, respectively.

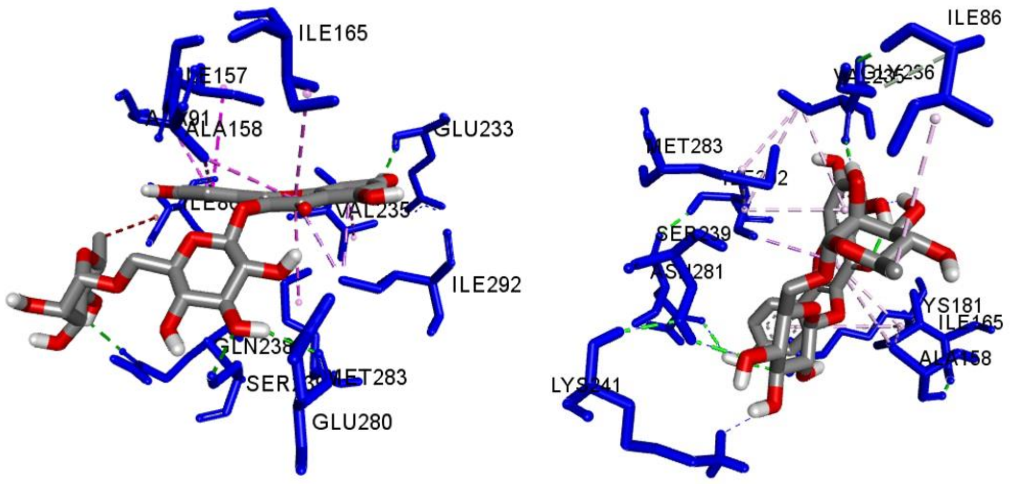

a) **b)**

Figure 1. a) Docked pose of rigid nicotiflorin (**3**) in the *Mt*PknG binding site showing molecular interactions - hydrogen-bonds as green dashed lines and hydrophobic bonds as pink/purple dashed lines- between (**3**) and *Mt*PknG, generated by BIOVIA Discovery Studio visualizer. **b)** Docked pose of flexible nicotiflorin (**3**) in the *Mt*PknG binding site showing molecular interactions - hydrogen-bonds as green dashed lines and hydrophobic bonds as pink/purple dashed lines- between (**3**) and *Mt*PknG, generated by BIOVIA Discovery Studio visualizer.

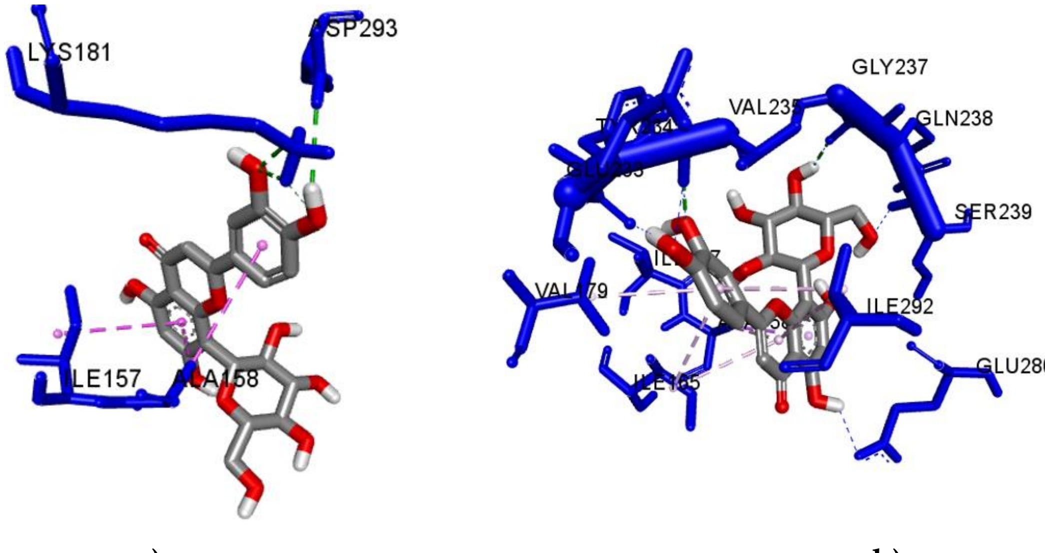

a) **b)**

Figure 2. a) Docked pose of rigid orientin (**4**) in the *Mt*PknG binding site showing molecular interactions - hydrogen-bonds as green dashed lines and hydrophobic bonds as pink/purple dashed lines- between (**4**) and *Mt*PknG, generated by BIOVIA Discovery Studio visualizer. **b)** Docked pose of flexible orientin (**4**) in the *Mt*PknG binding site showing molecular interactions - hydrogen-bonds as green dashed lines and hydrophobic bonds as pink/purple dashed lines- between (**4**) and *Mt*PknG, generated by BIOVIA Discovery Studio visualizer.

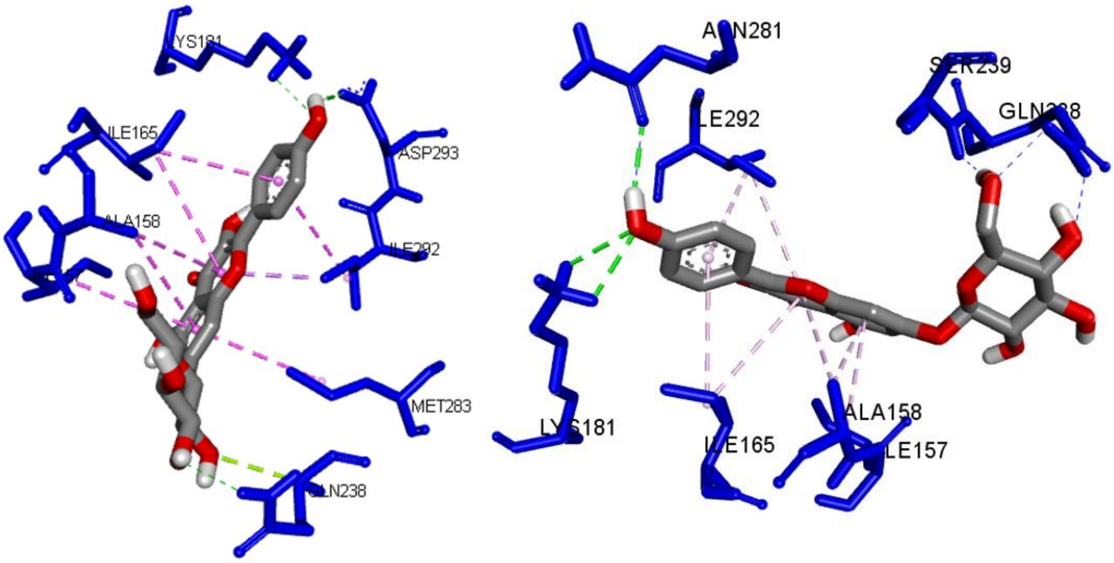

a) **b)**

Figure 3. a) Docked pose of rigid populnin (**5**) in the *Mt*PknG binding site showing molecular interactions - hydrogen-bonds as green dashed lines and hydrophobic bonds as pink/purple dashed lines- between (**5**) and *Mt*PknG, generated by BIOVIA Discovery Studio visualizer. **b)** Docked pose of flexible populnin (**5**) in the *Mt*PknG binding site showing molecular interactions - hydrogen-bonds as green dashed lines and hydrophobic bonds as pink/purple dashed lines- between (**5**) and *Mt*PknG, generated by BIOVIA Discovery Studio visualizer.

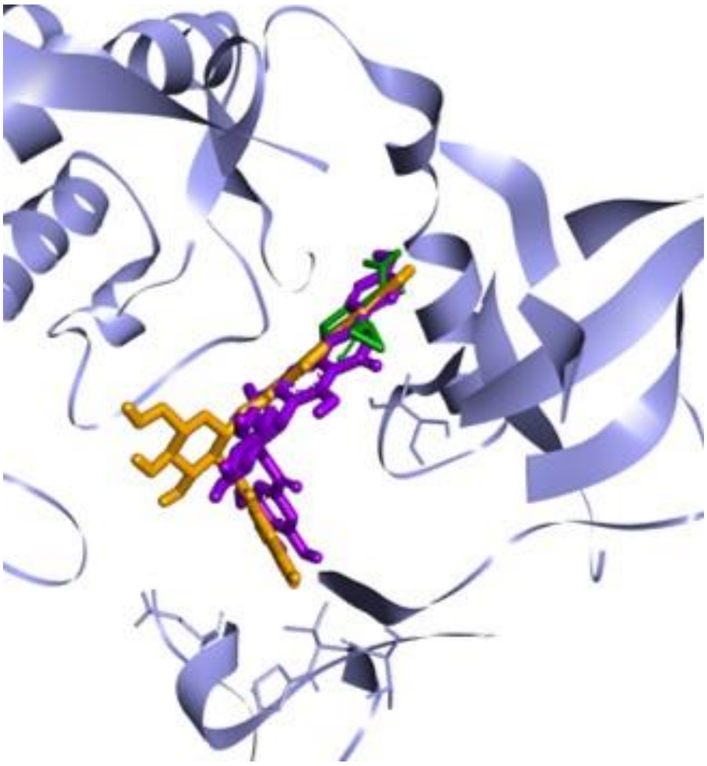

Figure 4. Overlay of the docked poses of the control inhibitor (green), isoorientin 2″-*O*-gallate (**1**) (yellow) and isovitexin 2″-*O*-gallate (**2**) (purple) in the *Mt*PknG binding site following rigid ligand docking, generated by BIOVIA Discovery Studio visualizer.
